# Evaluating Human Movement Coordination During Immersive Walking in a Virtual Crowd

**DOI:** 10.3390/bs10090130

**Published:** 2020-08-27

**Authors:** Alexandros Koilias, Michael Nelson, Sahana Gubbi, Christos Mousas, Christos-Nikolaos Anagnostopoulos

**Affiliations:** 1 Department of Cultural Technology and Communication, University of the Aegean, 81100 Mytilene, Greece; ctd17008@aegean.gr (A.K.); canag@ct.aegean.gr (C.-N.A.); 2 Department of Computer Graphics Technology, Purdue University, West Lafayette, IN 47907, USA; nelso430@purdue.edu (M.N.); sgubbi@purdue.edu (S.G.)

**Keywords:** virtual reality, movement coordination, virtual crowd, human–crowd interaction, immersive interaction, road crossing

## Abstract

This paper describes our investigation on how participants coordinate movement behavior in relation to a virtual crowd that surrounds them while immersed in a virtual environment. The participants were immersed in a virtual metropolitan city and were instructed to cross the road and reach the opposite sidewalk. The participants performed the task ten times. The virtual crowd that surrounded them was scripted to move in the same direction. During the experiment, several measurements were obtained to evaluate human movement coordination. Moreover, the time and direction in which the participants started moving toward the opposite sidewalk were also captured. These data were later used to initialize the parameters of simulated characters that were scripted to become part of the virtual crowd. Measurements were extracted from the simulated characters and used as a baseline to evaluate the movement coordination of the participants. By analyzing the data, significant differences between the movement behaviors of the participants and the simulated characters were found. However, simple linear regression analyses indicated that the movement behavior of participants was moderately associated with the simulated characters’ movements when performing a locomotive task within a virtual crowd population. This study can be considered as a baseline for further research that evaluates the movement coordination of participants during human–virtual-crowd interactions using measurements obtained by the simulated characters.

## 1. Introduction

We are commonly surrounded by and walk side-by-side with other people, and we coordinate our movements based on the people who surround us. Examples include dancing, playing team sports, and walking on sidewalks and through parades. According to previous studies conducted by Marsh et al. [1] and Jiang et al. [2], when two or more people perform an action with other people (e.g., walking toward a target position), one person’s actions mutually influence the actions of the other people, resulting in that particular group acting as a unit. Moreover, it has been found that people tend to synchronize their movements and their steps by following a tempo [3] when walking together [4,5].

This paper investigates participants’ movement coordination during immersive crowd interactions, a common type of interaction people encounter on a daily basis when walking in real environments. More specifically, one might want to virtually travel to a metropolitan city and take a short walk on a sidewalk to explore the surroundings. During this experience, the users of such an application might be surrounded by a virtual population. Therefore, it becomes vital to understand whether and how the virtual population affects a user that is walking in the virtual environment in order to design virtual reality experiences that better replicate such interactions with virtual pedestrians. In this study, participants were placed at a crosswalk in a virtual metropolitan city and were instructed to cross the road and reach the opposite sidewalk. A virtual crowd was also scripted to move in the same direction. During the road crossing, several measurements were collected to assess the movement behaviors of the participants. In addition, parameters such as the initial direction and the time step of the simulation in which the participant started walking toward the opposite sidewalk were collected and later used to simulate the virtual characters scripted to be part of the virtual crowd. For the simulated characters, all examined measurements were computed and used as the baseline to (a) investigate whether the participants’ movement behaviors differed from the movement behaviors of the simulated characters, and (b) examine the possible relationships between the movements of the participants and those of the simulated characters. The movement behaviors of the simulated characters were considered to be ideal, since they were scripted to become part of the moving crowd using Reynolds’ rules [6] on flocking behavior (separation, alignment, and cohesion).

Based on our implementation, we investigated how participants coordinate their movement behaviors when immersed in a virtual crowd interaction scenario. We would like to note that by following Reynolds’ rules [6] on flocking behavior, the simulated characters coordinated with the virtual crowd and became part of it. Thus, by exploring potential differences and associations between the participants’ and simulated characters’ movements, we explored whether the participants coordinated with the virtual crowd, and consequently, we investigated whether the human participants became part of it. Although we found significantly different movement behaviors for some of the participants’ measurements compared to those of the simulated characters, we were also able to identify significant relationships between the movement behaviors of the participants and those of the simulated characters. The results demonstrate how the participants coordinated their movements in accordance with the virtual crowd that surrounded them when performing a locomotive task, and whether the participants became members of the virtual crowd (acted as boids) by following it using movement behaviors similar to those the simulated characters were scripted to perform.

The remainder of this paper is arranged as follows. Section 2 covers the related work. Section 3 describes the methodology and implementation details of the experimental study. The results are presented in Section 4 and discussed in Section 5. Finally, the conclusions and future research directions are discussed in Section 6.

## 2. Related Work

Multiple studies have assessed human behavior and perception during various virtual-reality interaction tasks [7,8,9,10,11,12,13,14]. The popularity of virtual reality for performing experimental studies regarding perception, action, and behavior can be attributed to several factors. First, virtual environments are safe for the participants. Second, it is possible for all participants to be exposed to the same experimental conditions. Finally, the influences of external parameters are minimized, since the experiment is performed in a controlled lab space and because the participants wear a full-blind head-mounted display. Thus, it is easy to argue that virtual reality is a powerful tool that can be used for various studies related to human perception, activity, and behavior [15].

Most research in this field has investigated how human movement is affected by the virtual environment [16,17] or has examined human movement coordination and regulation during locomotive behavior in real or virtual environments [18,19,20]. Extensive research has been conducted on how humans coordinate and regulate their movement behavior according to others [21,22] or according to various walking tasks [17,19,23], and on how humans behave in teams and team sports [24,25]. In addition, various studies on movement regulation, such as following behavior [26,27], side-by-side walking [28], face-to-face walking [29], group formations [30], and collision avoidance [19,20], have also been explored.

Movement coordination and synchronization occur because of mirror neurons. According to the literature [31,32,33,34,35], the mirror neuron fires when a human acts or when a human observes an action being performed by another human. Thus, a neuron "mirrors" the behavior of the other human as if the observer were acting [36]. When we observe a human performing an action, which in our case it might also be a locomotive movement performed by virtual characters, we tend to perform the same actions ourselves. Humans tend to coordinate their actions according to the actions of others, even if the actions are not related to the task they are performing. A consequence of this is that such coordination might damage the performance of that assigned task [34,37,38]. Interestingly, it has also been found that when humans are able to predict the upcoming movements of others, this expectation activates the electrophysiological markers of motor preparation even before observing the expected movement [39,40,41]. Motion anticipation and preparedness are essential to various tasks, ranging from simple (walking) to more complex ones (dancing with a partner and interacting with teammates). To ensure movement coordination with others, the leader is responsible for modifying his/her actions to signal his/her intentions for action alteration to their partner [42,43]. Humans also tend to incorporate the dexterity of other humans into their planning strategy to achieve the required goal [22]. By doing so, we are able to regulate our movement in order to perform coordinated and synchronized actions with others.

Movement coordination and regulation have been studied in various domains. Such studies include real [44,45,46] and virtual reality [2,47,48] scenarios in which people coordinate while crossing an intersection in the presence of either a human or a virtual character [2,48]. It has been found [49,50,51] that when two humans who are part of a group are crossing a road, they become sensitive to the presence of the each other and tend to simultaneously change their decisions or their actions, compared to when they are crossing a road on their own. Based on a human–virtual-crowd interaction study, Warren [52] found that participants (followers), rather than keeping a constant distance from the leaders, matched the leaders’ velocity. Based on this finding, a dynamical model was also built of how a pedestrian aligns his or her motion with that of a neighbor and how these binary interactions are combined within a neighborhood of interaction. Rio et al. [53] investigated the neighborhood of interaction in a crowd—more specifically which neighbors influence a pedestrian’s behavior, how this depends on neighbor position, and how the influences of multiple neighbors are combined. They found that neighbor influence is linearly combined and decreases with distance, but not with lateral position.

It has been found that negative reactions are triggered when a participant’s personal space is violated by a virtual character [54,55]; therefore, participants try to maintain a greater distance when interacting with virtual characters, and this is especially true when interacting with realistic virtual characters [56]. Similarly, participants tend to maintain a greater distance when approaching virtual characters from the front compared to approaching them from behind [57]. Another study [18] has found that participants tend to follow a longer path around dense groups of virtual characters. These findings motivated us to further study human movement behaviors and to determine whether humans become part of the virtual crowd. Our work goes a step further by investigating how the participants coordinate their movement behavior when immersed in a virtual crowd. Therefore, instead of studying movement coordination with a single virtual character, we investigated movement coordination with a virtual crowd.

Among other human–crowd interaction studies, a virtual reality framework examining the human–virtual-crowd interaction and ways to improve the level of realism of simulation algorithms was developed by Olivier et al. [58]. Dickinson et al. [59] studied the effects of crowd density across three experimental conditions on the affective states and behaviors of participants. The results showed a significant increase in negative effects when interacting with a high-density crowd, measured using self-reported ratings. They also found significant differences in certain aspects of participant behavior using a video analysis technique. Rios et al. [60] studied the effect of crowd social behavior on human behavior when following others during a virtual reality evacuation scenario. The results of this study indicated that an accurate model has not yet been developed to determine under what circumstances and to what extent this behavior emerges. Finally, by simulating variations in a crowd’s speed, direction, and density, Nelson et al. [61] studied whether such variations can alter the movement behavior of the participants, and it was found that the density and speed of the crowd altered the speed and direction of the participants.

The current study is an extension of previous studies that have examined human movement coordination in virtual environments [2,18,19,20,62,63] and human–virtual-crowd interactions [59,61,64,65,66]. However, less attention has been paid to understanding how humans coordinate their movement when surrounded by a crowd. To the best of our knowledge, no prior study has evaluated the participants’ movement coordination in virtual environments against data obtained from simulated characters. Therefore, the evaluation of movement coordination through the use of simulated characters is the main contribution of this paper.

## 3. Materials and Methods

This section presents the methodology and the implementation details of the study.

### 3.1. Participants

The participants were recruited through class announcements and emails. The participant group was comprised of 80 undergraduate and graduate students. Of the participants, 22 were female and 58 were male. The students’ ages ranged from 19 to 37 years, with a mean of M = 22.51 (SD = 3.02). Of the sample, 43 participants had prior experience with virtual reality. All participants were volunteers, and no compensation was offered. None of the participants reported motion sickness or other types of cybersickness. Most previous studies investigating human movement behavior in virtual reality environments used small sample sizes [2,20,62] and an increased number of trials that each participant performed to smooth out the captured movement behavior. It should be noted that in this study, we took into account the recommendations on sample size for linear regression made by Dupont et al. [67]; therefore, a larger sample size was recruited to ensure the reliability of the results, and multiple trials (as in Rahimian et al. [68] and Jiang et al. [2]) were performed by participants to smooth out the captured movement behavior.

### 3.2. Setup and Virtual Reality Application

The research team conducted the virtual reality study by using our department’s motion capture studio. The studio was eight meters long and eight meters wide with a ceiling height of four meters. This studio was appropriate for the experimental study, as it contained no obstacles other than a computer desk and a couple of chairs. The HTC Vive Pro head-mounted display device was used to project the virtual reality content, an Xsens inertial motion capture system was used to capture the movement of the participants, and the MSI VR One backpack computer (Intel Core i7, NVIDIA GeForce GTX1070, 16GB RAM) was used to run the application. Note that we decided to conduct our study using a consumer-grade head-mounted display, as virtual reality users are most likely to use consumer-grade ones to experience immersive walking in their own home setup.

**Figure 1:**
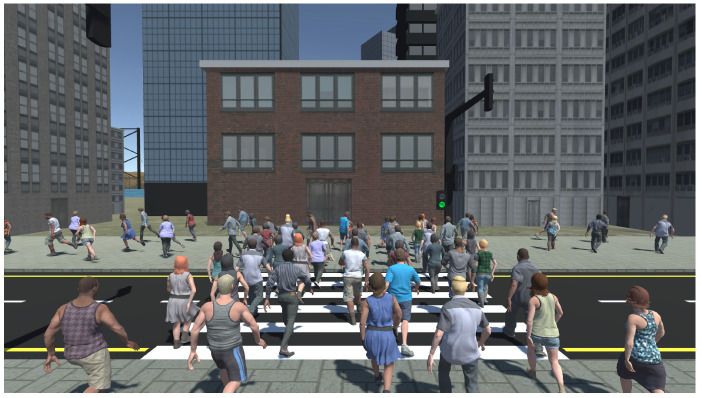
The virtual crosswalk used in this study and the virtual crowd that was scripted to move toward the opposite sidewalk.

**Figure 2:**
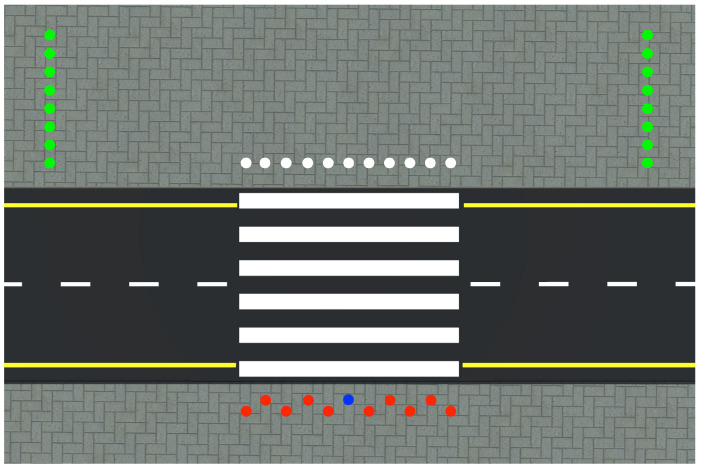
The positions the virtual pedestrians were initialized on the sidewalk are indicated by red circles, and they were scripted to reach one of the target positions indicated by white circles. Then the virtual pedestrians were asked to reach one the of green circles to alleviate congestion on the sidewalk. The participants were placed on the blue circle. All circles were not visible during the study. The blue circle corresponded to a marked location in the motion capture studio.

The application used for this study was developed in the Unity game engine version 2019.1.4. A virtual metropolitan city was designed in 3ds Studio Max and imported to the Unity game engine to be used for the study. The virtual environment (crosswalk) used for this experiment is illustrated in Figure 1. Each participant was placed on the edge of a crosswalk in the virtual metropolitan city. For the virtual crowd simulation, thirty virtual pedestrians (15 male and 15 female) were designed in Adobe Fuse and rigged using Adobe Mixamo. The Mecanim animation engine of Unity was used to animate the pedestrians along with the motion data downloaded from the Unity Assets Store. The virtual pedestrians were generated on a constant basis one in every second from ten spawn points placed behind the participant’s position in the virtual environment (see Figure 2). Each character’s crossing was repeated multiple times.

**Figure 3:**
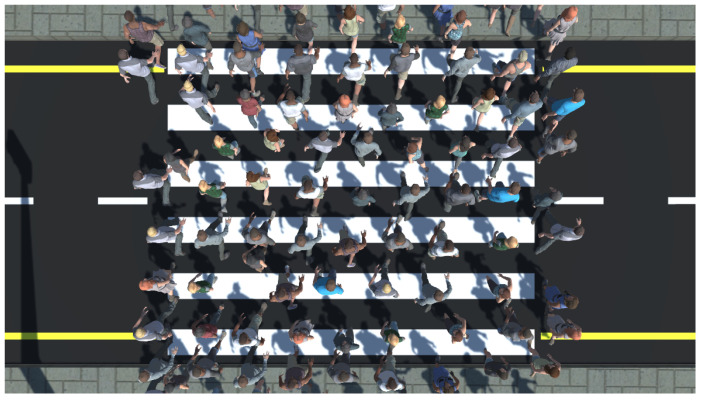
The medium size density model (1.5 pedestrians per square meter) that was used in the conducted experimental study.

The virtual pedestrians (virtual crowd) were pre-scripted to cross the road and reach the opposite sidewalk which was consistent with the direction of the participant. After reaching the opposite sidewalk, each pedestrian was scripted to move to another location in the virtual environment to alleviate congestion on the opposite sidewalk. We decided to simulate a crowd with a medium density (1.5 pedestrians per square meter, see Figure 3), as proposed by Still [69], since we did not want to violate the personal space of the participants, which might have triggered unnecessary alterations in their behavior, as has already been found [55,61].

Sunlight was used to light the scene, and audio effects mimicking the sounds of a metropolitan city full of pedestrians were used to enhance the sensation of being within the virtual environment. Note that no self-avatar was used to represent the participants within the virtual environment. This was because a prior study has indicated that a self-avatar may alter the movement behavior of the participants [19]. The opposite sidewalk was seven meters away from the position of the participant, which means that the participants were asked to walk this distance.

### 3.3. Measurements

To analyze and evaluate human behavior, behavioral, motion, and electrophysiological recording techniques are typically used [19,39,40,70,71,72,73]. In this study, to determine how participants coordinated their movement compared to simulated characters, we computed measurements related to the task and the objectives of the experiment. To do so, we used a motion capture system, and the captured motion of the participants was downsampled in one hundred equidistant points, as in [17]. Then, the data for each measurement were extracted. The included measurements were as follows:

- **Speed:** The average speed of the participant’s walking motion when crossing the virtual crosswalk. The speed was measured in meters/second.
- **Time:** The time a participant needed to cross the virtual crosswalk (reach the opposite sidewalk). The time was measured in seconds.
- **Length:** The total trajectory length (covered distance) of the participants. The length of the captured trajectory was measured in meters.
- **Direction:** The average absolute *y*-axis rotation on the (x, z) plane of the participant when walking toward the opposite sidewalk. Direction was measured in degrees. Zero degrees indicated that the participant was moving parallel to the segment that connected his/her initial position and the forward position on the opposite sidewalk.
- **Smoothness:** The smoothness was computed as the average flicker of the trajectory, as in [74]. Low flicker values denoted a smoother trajectory. The smoothness was measured in meters.
- **Distance from nearby pedestrians:** We computed the average distance from the closest four virtual pedestrians in front of the participant when moving toward the opposite sidewalk. The chosen virtual pedestrians were the same for the participants and the simulated characters and did not change during the walking task. Note that for each trial, different nearby virtual pedestrians were chosen. The distance from nearby virtual pedestrians was measured in meters.

### 3.4. Procedure

**Figure 4:**
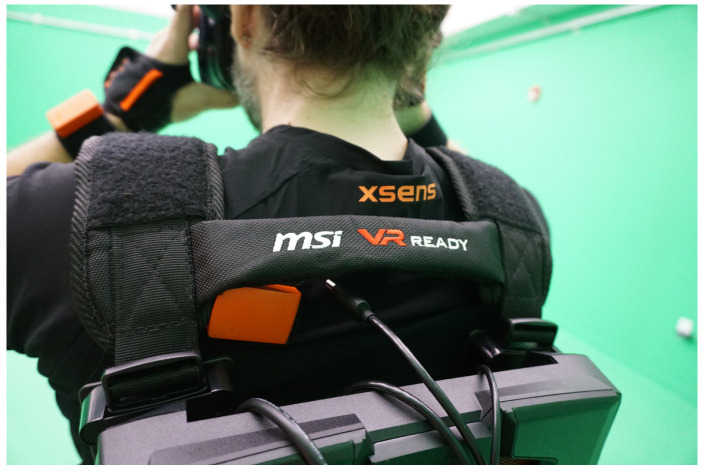
The participant wearing all devices (MSI VR One backpack computer, HTC Vive head-mounted display, and Xsens motion capture system) used for this study.

Once the participants arrived at the motion capture studio, the experimenter provided information about the project, and the participants were asked to sign the provided consent form that was approved by the Institutional Review Board of ANONYMIZED University. Then, the participants were asked to complete a demographic questionnaire. In the next step, the experimenter helped the participants wear the Xsens motion capture system, the backpack computer, and the head-mounted display. The participants were then asked to take a short walk within the motion capture studio to ensure they were comfortable when wearing all the devices. Figure 4 depicts a participant wearing these devices.

After becoming comfortable and familiarized with the equipment, the experimenter asked the participants to move toward a marked location in the motion capture studio and face the opposite direction. Once the participants landed on the marked position, they were informed that once the application started, they would be placed in a virtual metropolitan city and would have to cross the virtual crosswalk by moving toward the opposite sidewalk. The participants were informed that they would be surrounded by a virtual population and that it was up to them to decide whether they should start walking. The participants were also informed that once they reached the opposite sidewalk, a "beep" sound would signal them to stop walking. The participants were informed that they would cross the virtual crosswalk to reach opposite sidewalk ten times.

Once the participants finished each trial, they were asked to take off the head-mounted display and return to the marked location on the other side of the room. The participants were told they could have breaks between trials of the conditions if needed and that they had full permission to leave at any time. They were also told that they would be informed when the experiment had ended. After the tenth trial of the study, the experimenter helped the participants remove the head-mounted display, backpack computer, and motion capture system. Then, the participants were asked to describe their experiences and provide feedback on their movement behavior within the virtual environment by writing on a blank sheet which was provided to them. Finally, they were informed that the experimenter would be willing to answer questions about the study. None of the participants spent more than 40 min in the motion capture studio.

### 3.5. Simulated Characters

In this paper, we evaluated whether the participants coordinated their movement similarly to a simulated characters, and consequently, whether they became members of the virtual crowd (acted as boids). In addition to the measurements that were collected to analyze the movement behavior of the participants, data related to the timestep in which the participants started walking toward the opposite sidewalk and the direction of the participants at that time were also collected. These data were essential to initializing the parameters of the simulated characters that were used to evaluate the movement behavior of the participants.

The simulated characters were scripted to follow Reynolds’ rules [6] on flocking behavior:

- **Separation:** the simulated characters should steer to avoid crowding nearby virtual pedestrians.
- **Alignment:** the simulated characters should steer toward the average heading of nearby virtual pedestrians.
- **Cohesion:** the simulated characters should steer toward the average position of nearby virtual pedestrians.

The movement behavior of simulated characters is highly influenced by the movement behavior of nearby virtual pedestrians (the character that compose the virtual crowd); however, the movement of the virtual pedestrians was not affected by the movement of participants and the simulated characters, that were later used to evaluate the movement coordination of participants. Based on the nearby virtual pedestrians, our system computed the updated position of the simulated virtual character at a frame-by-frame rate until that character would reach the opposite sidewalk. Two additional parameters were implemented. First, the closest distance between two virtual pedestrians was chosen to be the boundaries of the close phase of the personal space (76 cm) according to the proxemics model [75,76]. Moreover, we set the crowd’s speed to not exceed 1.2 m/s. This choice was based on the U.S. Manual of Uniform Traffic Control Devices [77], which defines that the normal walking speed of humans has been estimated to be 1.2 m/s.

## 4. Results

This section presents the results of the study. All the analyses were performed using IBM SPSS version 24.0 [78] software. Paired-samples t-tests were used to determine differences between the measurements obtained from the participants and those obtained from the simulated characters. Then, simple linear regressions were used to explore how the movement behavior of the participants is associated with the movement behavior of the simulated characters. The normality assumption of the collected data was evaluated graphically using Q–Q plots of the residuals [79]. The Q–Q plots indicated that the obtained data fulfilled the normality criteria. Finally, a p < 0.05 value was deemed statistically significant.

### 4.1. Movement Behavior Differences

**Figure 5:**
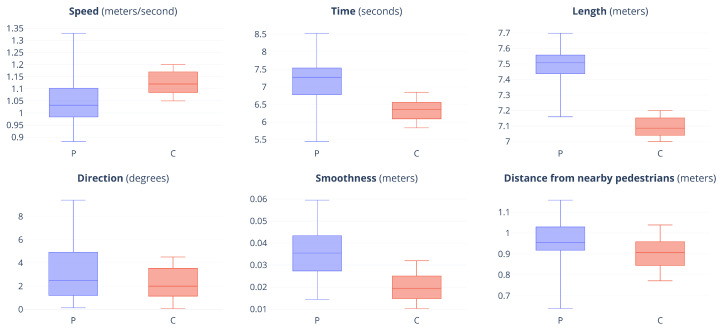
Boxplots of the results of all measurements for the participants (P) and simulated characters (C). Boxes enclose the middle 50% of the data. The median is denoted by a thick horizontal line.

The pairwise relationships between the measurements obtained from the participants and those obtained from the simulated characters were compared using paired-samples t-tests. Boxplots of all measurements are shown in Figure 5. We identified a significant difference between the speed (meters/second) of the participants (M = 1.05, SD = 0.10) and the simulated characters (M = 1.12, SD = 0.05); t(79) = −7.111, p < 0.001. The difference in time (seconds) among the participants (M = 6.93, SD = 0.62) and the simulated characters (M = 6.45, SD = 0.32) was also significant; t(79) = 8.269, p < 0.001, p < 0.001. The trajectory length (meters) was significantly different among the participants (M = 7.52, SD = 0.076) and the simulated characters (M = 7.09, SD = 0.06); t(79) = 22.716, p = 0.001. The direction (degrees) was not significantly different among the participants (M = 3.12, SD = 2.49) and the simulated characters (M = 2.70, SD = 1.13); t(79) = 1.879, p = 0.064. The smoothness (meters) was significantly different among the participants (M = 0.04, SD = 0.01) and the simulated characters (M = 0.02, SD = 0.01); t(79) = 8.738, p < 0.001. Finally, the distance from nearby pedestrians (meters) was significantly different among the participants (M = 0.96, SD = 0.08) and the simulated characters (M = 0.90, SD = 0.06); t(79) = 9.543, p < 0.001.

### 4.2. Movement Behavior Relationship

The relationship between the measurements obtained from the participants and those obtained by running the simulations was evaluated by running simple linear regressions. We conducted linear regression using the measurements extracted from our participants as dependent variables, and the measurements extracted from the simulated characters as independent variables. Detailed results are provided in the regression table (see Table 1). We used linear regression for three reasons. First, prior studies [61,80] found that the movement behavior of participants can be affected by the movement behavior of the virtual crowd. Second, because in this implementation the virtual crowd was scripted to move independently of participants, the movement behavior of participants could not influence the movement behavior of the virtual crowd. Third, linear regression was used to measure the extent of the linear relationship among the examined variables.

The results indicated that the participants’ **speed** (meters/second) was significantly associated with the simulated characters’ speed (F(1,78) = 31.284, p < 0.001, $R^{2}\;=\;0.286$). The results also showed that the participants’ **time** (seconds) was significantly associated with the simulated characters’ time (F(1,78) = 37.626, p < 0.001, $R^{2}\;=\;0.325$). The trajectory **length** (meters) of the participants was not associated with the trajectory length of the simulated characters (F(1,78) = 0.396, p = 0.531, $R^{2}\;=\;0.005$). The participants’ **direction** (degrees) was significantly associated with the simulated characters’ direction (F(1,78) = 52.922, p < 0.001, $R^{2}\;=\;0.404$). In addition, there was not a significant correlation of trajectory **smoothness** (meters) between the participants and the simulated characters (F(1,78) = 3.130, p = 0.081, $R^{2}\;=\;0.039$). Finally, the results showed that the participants’ **distance from nearby pedestrians** (meters) was significantly associated with the simulated characters’ distance from nearby pedestrians (F(1,78) = 43.635, p < 0.001, $R^{2}\;=\;0.359$).

## 5. Discussion

The objective of this study was to determine how participants coordinate their movement behavior when crossing a road to reach the opposite sidewalk while immersed in a virtual metropolitan city co-occupied with a virtual crowd population moving in the same direction. The participants began each trial surrounded by a virtual crowd. They were asked to cross the road and reach the opposite sidewalk but were not given additional instructions on following the movement behavior of the nearby virtual pedestrians. The collected data were analyzed to determine whether the participants became part of the virtual crowd, thereby becoming boids (matching the movement behavior of the simulated characters).

The results indicated that when walking within a virtual crowd in an immersive environment, the participants showed a clear disinclination to performing the walking task in the same way the simulated characters did. The participants tended to move slower and therefore required more time to accomplish the task, followed longer paths, performed less-smooth motions, and allowed more distance between themselves and the nearby virtual pedestrians. These findings suggest that the participants performed their movements differently from the simulated characters. However, when examining the relationships between the movement behaviors of the participants and the simulated characters, the speed, time, direction, and distance from nearby pedestrians were significantly associated.

By combining the results obtained through the comparisons and regression analyses, it can be said that the virtual crowd did influence the movement behaviors of the participants, since it appears that the participants associate their movement with that of the virtual crowd that surrounded them. However, the influence of the crowd on the participants’ movement was not enough to make the participants follow the movement of the crowd with the exact same pattern compared to the simulated characters. This finding might be related to several other studies that have examined concurrent and joint human actions [81,82,83,84]. To explain this finding, we base our discussion in perception-action-related studies that concern human movement behavior. According to Gibson [85], when humans walk with each other, they take time to process their course of action and they initiate their walking only after they feel safe enough to merge between themselves and the nearby humans. Moreover, Brass et al. [37] found that the observation of a movement affects the execution of a similar movement, and Watanabe [86] indicated that humans tend to modify their movement, since the speed of other humans may influence the timing of the movement execution. That might explain why the participants did not highly coordinate their movements and why they decided to follow the movement of the virtual crowd in a moderate degree.

The participants were highly immersed in and engaged with the virtual environment and were highly focused on accomplishing the given task appropriately. This high level of immersion and engagement can be explained through several factors. First, the participants were wearing a full-blind head-mounted display, so no external parameters existed to interfere with their sensations. Second, the participants performed a walking motion in the virtual environment. This is important, since this action triggers the sensory motor system (haptic, proprioceptive, and vestibular systems) of humans that supports locomotion. This combination of factors created a compelling experience.

Moreover, the participants were not asked to follow or imitate the movement behavior of the virtual crowd population. The participants’ decisions and movement behaviors were made autonomously, which means that it was they who decided how they should move and how they should try to coordinate their movement with the nearby pedestrians. Interesting questions could be raised about the decision made by participants to regulate their movement behavior when moving within a virtual crowd. Research in the field of psychology [87] has examined the perception of the movement of others and provides interesting insights on how to interpret such decisions. It has been found that by observing the motions of others, it is possible to extract useful information about the actions, moods, and their intentions, which renders this observation into a critical channel of communication [88,89,90]. Moreover, Adams [91] has addressed the nature and contribution of sensory feedback in movement coordination (timing and positioning) and said that there is no a priori reason why feedback from any sensory system cannot inform about movement. This suggests that the participants may have responded to the movement behavior of the nearby virtual pedestrians after assessing their intention of how and when to move toward the opposite sidewalk in the virtual environment.

Although we were able to find several significant relationships between the movement behaviors of the participants and the simulated characters, we also found significant differences. This means that the participants walked differently than the simulated characters. A couple of participants mentioned that the virtual reality equipment they were wearing made them decide to move slower because they were concerned about damaging it. Other participants reported that they did not feel comfortable within the virtual population. Consequently, this might have compelled them to try to avoid nearby pedestrians and deviate from the ideal path, thus following longer routes. Similarly, participants reported that they were trying to follow clear or less dense areas so they could move more freely. Furthermore, some of the participants expressed that they became confused with the absence of a self-avatar, and this absence made them unaware of their exact position, their boundaries, and whether they had collided with the virtual pedestrians. Finally, some of the participants reported that they moved slower because they did not have prior experience moving within virtual environments.

Based on the literature, it has been found that humans’ fear of colliding with obstacles (including walls) in real environments when immersed within a virtual environment often reduces their natural locomotion behavior and gait, consequently altering their movement behavior in virtual environments [92]. Moreover, it has been found that aesthetic mismatching between the real and visual environment also affects the movement behavior of participants [17,93]. Although the real environment was free of obstacles, and the virtual environment was structured in a similar way to the real environment (meaning that the virtual environment was free from obstacles and with a flat terrain), both fear of collision and aesthetic mismatch might have influenced the movement behavior of the participants.

It is worth also noting how the simulated characters were scripted to move toward a particular direction. In our case, even if the simulated virtual character had self-awareness of its relevant position, direction, speed, and collision with the virtual pedestrians, it would move to the opposite sidewalk without being influenced by external parameters and visual stimuli, as our participants were. This might partially explain why the trajectories from the simulated characters were smoother than the trajectories of the participants. Another factor for this reduced smoothness could be that the signal from the motion capture system may be affected by the presence of unwanted noise.

When comparing the movement behavior of humans with the movement behavior of simulated characters, such evaluations might be problematic since they assess human behavior against computer-generated behaviors which are highly constrained and scripted so that they strictly follow rules without deviations. It is therefore assumed that data-driven crowd simulation techniques [94,95,96] might be more suitable for synthesizing the movement of virtual pedestrians, since the extracted measurements will be closer to real-life measurements. However, further experimentation is needed to arrive to more substantial conclusions. Overall, even if the use of measurements obtained from the simulated characters and from the movement behavior of the participants is considered too constrained due to the nature of the simulation, useful information regarding human movement behavior could still be obtained.

## 6. Conclusions and Future Work

This study investigated the movement coordination of participants when immersed within a virtual crowd population. The results indicate that the participants’ movements were associated with the simulated characters’ movements. The strength of this relationship is moderate, especially in the cases of direction and distance from nearby pedestrians. The study shows that the influence of the crowd on the participants’ movements was not enough to make the participants coordinate their movements to be similar to the simulated characters, follow the movement of the crowd, and therefore become a part of it.

Based on our findings, several interesting insights about the participants’ movement behavior and coordination within a virtual crowd population were obtained. This paper demonstrates that immersive human–crowd interaction scenarios can be used to study the actions and decision-making processes of humans, since the developed experimental conditions can be controlled by the experimenter and replicated by other researchers. Moreover, participant movement can be captured efficiently, which is critical for such studies. Studying human movement behavior and coordination when the participants are immersed in virtual crowds is an interesting direction for studying perceptual-motor tasks that require decision-making and action-planning. However, the concerns raised from this study should be considered in future studies. Finally, the findings of this research along with future studies should prove to be invaluable resources when developing virtual reality applications and games in which virtual reality users are placed within moving virtual crowds.

This study has attempted to set the foundation for future studies evaluating human–virtual-crowd interactions in immersive virtual environments based on data obtained from simulated characters. In future work, we will explore the interaction effects of crowd movement variations (speed, direction, and density) on human movement behavior, the effects of virtual pedestrians’ appearance and motion assigned to a crowd on participants’ emotional reactivity [97], the effects of tactile feedback during immersive walking in virtual crowds [98], and the effects of self-avatars on human movement and flocking behavior. We also plan to collect other forms of data, such as eye tracking, electrodermal activity, and subjective ratings [73,99], to study the interactions between humans and virtual crowds. Finally, experimentation with data-driven techniques for simulating the movement of virtual pedestrians will also be explored.

## Figures and Tables

**Table 1:** Regression table for all examined measurements. The unstandardized coefficient (*B*), the standard error for the unstandardized coefficient (*SE* *B*), the standardized coefficient (*β*), the *t*-test statistic (*t*), and the probability value (*p*).

	*B*	*SE B*	*β*	*t*	*p*
**Speed**	1.146	0.205	0.535	5.593	0.001
**Time**	1.106	0.180	0.570	6.134	0.001
**Length**	−0.148	0.234	−0.071	−0.630	0.531
**Direction**	0.743	0.102	0.636	7.275	0.001
**Smoothness**	0.176	0.099	0.196	1.769	0.081
**Distance from nearby pedestrians**	0.674	0.098	0.599	6.606	0.001
